# Substance use and mental health factors associated with self-reported higher risk cannabis use among people with HIV screened in primary care

**DOI:** 10.21203/rs.3.rs-4415444/v1

**Published:** 2024-05-29

**Authors:** M.N. Mian, V. Sarovar, T. Levine, A. Lea, A. Leibowitz, M. Luu, J. Flamm, C. B. Hare, M. Horberg, K. C. Young-Wolff, K. T. Phillips, M. J. Silverberg, D. D. Satre

**Affiliations:** 1 Division of Research, Kaiser Permanente Northern California, Pleasanton, California, Pleasanton CA; 2 Department of Psychiatry and Behavioral Sciences, Weill Institute for Neurosciences, University of California, San Francisco, CA, USA; 3 Kaiser Permanente Oakland Medical Center, Oakland, CA, USA; 4 Kaiser Permanente Sacramento Medical Center, Sacramento, CA, USA; 5 Kaiser Permanente San Francisco Medical Center, San Francisco, CA, USA; 6 Mid-Atlantic Permanente Research Institute, Kaiser Permanente Mid-Atlantic States, Rockville, MD, USA; 7 Center for Integrated Health Care Research, Kaiser Permanente Hawaii, Honolulu, HI, USA; 8 Department of Health Systems Science, Kaiser Permanente Bernard J. Tyson School of Medicine, Pasadena, CA, USA

**Keywords:** cannabis, marijuana, HIV, primary care

## Abstract

**Background:**

While cannabis use is prevalent among people with HIV (PWH), factors associated with higher-risk use require further study. We examined factors associated with indicators risk for cannabis use disorder (CUD) among PWH who used cannabis.

**Methods:**

Participants included adult (≥18 years old) PWH from 3 HIV primary care clinics in Kaiser Permanente Northern California who reported past three-month cannabis use through the computerized Tobacco, Alcohol, Prescription medication, and other Substance use (TAPS) screening. Primary outcome was TAPS cannabis score (range 1–3), categorized as any use (1) and higher risk for CUD (≥2). Measures included sociodemographics (age, sex, race, neighborhood deprivation index [NDI]), Charlson Comorbidity Index (CCI), HIV RNA, CD4 cell counts, higher risk tobacco use (TAPS tobacco score≥2), depression, and anxiety symptoms. Unadjusted and multivariable logistic regression examined factors associated with higher risk for CUD.

**Results:**

Of the complete sample (*N*=978; 94.1% Male; 58.3% White; Age _Mode_=51–60), 35.8% reported higher risk for CUD. Unadjusted models indicated younger age, Black race, higher CCI, depression, anxiety, and higher risk tobacco use were associated with higher risk, while only Black race (OR=1.84, 95% CI[1.29, 2.63]), anxiety (OR=1.91, 95% CI[1.22, 2.98]), and higher risk tobacco use (OR=2.27, 95% CI[1.47, 3.51]) remained significant in the multivariable model.

**Conclusions:**

Black race, anxiety and tobacco use, but not HIV clinical markers, were associated with higher risk for CUD among PWH. Clinical efforts to screen and provide interventions for preventing CUD alongside anxiety and tobacco use among PWH should be evaluated.

## Introduction

Cannabis use is prevalent in the United States, particularly among people living with HIV (PWH) Compared with 45% of adults without HIV, a nationally representative study found that 77% of PWH reported lifetime cannabis use [1]. Another study of over 10,000 PWH drawn from the Center for AIDS Research Network of Integrated Clinical Systems found that 31% met criteria for Cannabis Use Disorder (CUD), defined as problematic cannabis use resulting in functional impairment [2].

These high rates of cannabis use are concerning given negative consequences associated with use [3], [4]. While factors typically associated with any use are known, further research is required to determine which factors predict higher risk cannabis use among PWH. For example, PWH might have higher risk for CUD given the increased prevalence of mental health concerns in this population, although associations between cannabis use and depression and anxiety symptoms among PWH are not consistent [5]. HIV viral control may be another important factor related to cannabis use, but associations between HIV markers (i.e., HIV RNA and CD4) and cannabis use are also mixed [6]. Furthermore, the assessment of cannabis use severity in conjunction with medical and psychiatric factors is often lacking in existing studies. Standardized clinical assessments of cannabis use severity within a healthcare setting could provide important insights about the links between HIV markers, medical, and psychiatric factors, and cannabis use among PWH.

Identification of PWH at elevated risk for CUD requires further elucidation. The present study aimed to examine predictors of higher risk for CUD among PWH who reported recent cannabis use on a digital screening tool in HIV primary care settings in an integrated healthcare system. First, we developed a descriptive profile of PWH who reported any cannabis use in the prior three months. Second, we examined whether socio-demographic factors, other substance use (tobacco, alcohol), depression, anxiety, and HIV viral control were associated with being at higher risk for CUD at the time of screening.

## Methods

### Study design and sample

This study was based in Kaiser Permanente Northern California (KPNC), an integrated healthcare delivery system providing primary and specialty care to over 4.5 million people, including approximately 5,000 PWH across the three largest KPNC HIV primary care clinics in Oakland, San Francisco, and Sacramento. The Promoting Access to Care Engagement (PACE) trial examined mental health, substance use, and treatment outcomes for PWH following electronic screening combined with behavioral interventions in the three clinics. The study protocol was published previously [7]. All procedures were in accordance with and approved by the KPNC and University of California, San Francisco Institutional Review Boards, including waiver of informed consent to examine participant medical records.

The study sample included adult PWH who reported cannabis use in the prior three months based on screening delivered through the KPNC patient portal (KP.org) in the two weeks prior to an outpatient primary care appointment or during that appointment via clinic tablets between October 30, 2018 and July 17, 2020. Questionnaire results were automatically uploaded into the electronic health record (EHR) and available to clinicians during visits.

### Measures

The primary study outcome was screening for higher risk for CUD, identified with the publicly available Tobacco, Alcohol, Prescription medication, and other Substance use (TAPS) Tool, a computerized, self-administered instrument validated for use in primary care settings [8]. TAPS substance use scores range from 0–4 for alcohol and 0–3 for tobacco and cannabis. Participants who reported any tobacco, alcohol, or cannabis use in the past three months received a respective TAPS score of 1, and a TAPS score of ≥2 was considered higher risk for SUD for that substance, per TAPS scoring guidelines. Participants who used cannabis in the past three months answered follow-up items: “In the past 3 months, have you had a strong desire or urge to use marijuana at least once a week or more often?” and “In the past 3 months, has anyone expressed concern about your use of marijuana?”.

Demographic variables were collected through the EHR, including age at screening, sex, race/ethnicity, and neighborhood deprivation index (NDI), which is a census-informed indicator of socioeconomic status [9]. Comorbidities were assessed by the Charlson Comorbidity Index (CCI), extracted from EHR and modified to remove HIV/AIDS from the index [10]. HIV RNA (log_10_ transformed) and CD4+ T cell count (CD4; cells/μL) within six months of screening were also collected from the EHR and the KPNC HIV Registry [7].

SUD, depression, and anxiety disorder diagnoses were based on ICD-10 criteria during the period 12 months prior to screening and were extracted from EHR. Depression and anxiety symptoms were assessed with the Patient Health Questionnaire (PHQ-9) and General Anxiety Disorder (GAD-2), respectively [11], [12] concurrent with TAPS administration. Positive depression score was defined as a PHQ-9 score ≥10 and anxiety as a GAD-2 score ≥3 [12], [13].

### Data Analytic Plan

First, we performed descriptive analyses and compared PWH with self-reported higher risk for CUD (Cannabis TAPS score=2+) and those reporting any past three-month use (Cannabis TAPS=1). Second, we conducted unadjusted logistic regression models for each predictor and the outcome of scoring at higher risk for CUD. Finally, significant unadjusted predictors were included in a multivariable regression model. Analyses were conducted on SPSS v. 29.

## Results

### Study Sample

Adult PWH completed 3,903 questionnaires during the study period. Screenings were excluded for: being associated with cancellations or no-show visits (*n*=6); having no associated eligible visit (*n*=68); and repeated screenings (*n*=1000). Finally, PWH not reporting recent cannabis use at screening (*n*=1851) were removed, leaving 978 unique PWH.

The majority of participants were male (sex at birth; 94.1%) and the most common age of participants was 51–60 years old (31.3%; Table 1). More than half of participants were White (58.3%). Participants were distributed across levels of neighborhood deprivation, with slightly more participants in the least deprived category. Average log10 HIV RNA was 1.79 (*SD*=0.52) and 94.3% had HIV RNA levels <200 copies. Average CD4 cells per *u*L was 666.1 (*SD*=209.2). On the depression screen (PHQ-9), 15.9% of participants scored positive (10+), while on the anxiety screen (GAD-2), 16.2% scored positive (3+). PWH had prior depression (20.5%), anxiety (18.5%), and SUD diagnoses (19.2%), and 27.8% and 10.3% of participants reported higher risk alcohol and tobacco use, respectively.

Compared with those reporting any past three-month use, those at higher risk for CUD were also majority male and White (Table 1), but had a greater frequency of Black PWH and a lower frequency of individuals in the least deprived category of the NDI. Those at higher risk for CUD were also more likely to screen positive for depression, anxiety, and tobacco use, and have prior diagnoses for depression, anxiety, and SUD.

### Regressions for Higher Risk for CUD

In unadjusted models (Table 2), significant factors associated with higher CUD risk included: age 17–30 years (compared with 31–40; odds ratio (OR)=1.79; 95%CI=1.02, 3.15); Black race (compared with White race; OR=2.03; 95%CI=1.46, 2.83); higher CCI score (OR=1.10; 95%CI=1.01, 1.20) per 1 point higher; anxiety (OR=1.96; 95%CI=1.39, 2.77); depression (OR=1.50; 95% CI=1.06, 2.12); and higher risk tobacco use (OR=2.47; 95%CI=1.63, 3.74). In the multivariable model, PWH who were Black (compared with White; OR=1.84, 95%CI=1.29, 2.63), PWH with anxiety (OR=1.91, 95%CI= 1.22, 2.98) and higher risk tobacco use (OR= 2.27; 95%CI=1.47, 3.51) had greater odds for higher risk for CUD. Depression, CCI and age were not associated with reporting higher risk for CUD in the multivariable model.

## Discussion

The present study aimed to identify factors associated with higher risk for CUD among PWH reporting recent cannabis use on a digital screening tool in primary care. Our study found that 35.8% of the sample were at higher risk for CUD. Several factors were independently associated with higher risk for CUD, including Black race, anxiety, and higher risk tobacco use.

The finding that race/ethnicity was a significant predictor of CUD risk has important implications. Disparities in HIV care for Black individuals [14] and the increased risk for cannabis-related consequences among Black individuals in the general population [15] are previously documented. Our results similarly suggest that Black PWH might be particularly at risk for consequences related to cannabis use, and that both screening and intervention efforts could be leveraged to address potential disparities in risk and health outcomes.

Our study also found that PWH aged 17–30 were at greater CUD risk than older PWH in the unadjusted model. Age is an important factor among PWH who use cannabis. While cannabis use is prevalent among young adults generally, recent trends show more older adults are initiating cannabis use, particularly to address aging-related health concerns such as pain [16]. Though older PWH may benefit from anti-inflammatory effects of cannabis use [17], further work is needed to address the implications of cannabis on HIV status and age, given the clinical implications of poorer treatment adherence among younger PWH broadly [18].

Mental health problems might also be impacted by cannabis use among PWH [3]. In our study, depression and anxiety were associated with greater odds of higher risk for CUD in unadjusted models, although only recent anxiety symptom score was significant after adjustment for other factors. PWH may use cannabis to mitigate anxiety [19], though additional research is needed to better understand the short-term and long-term consequences of cannabis and anxiety for PWH. Screening of mental health symptoms, particularly in a primary care setting that can be sensitive to changes over time, can provide opportunities for intervention and may prevent worsening concerns related to cannabis use among PWH.

Relatedly, we found that higher risk tobacco use was associated with higher risk for CUD. Tobacco is highly prevalent among individuals with HIV [20] and co-use of cannabis and tobacco is common in general [4]. Interventions to address smoking among PWH may be particularly helpful for reducing use and mitigating harms related to both cannabis and tobacco use.

Notably, we did not find associations between HIV indicators (HIV RNA levels, CD4 counts) and CUD risk. One study found that all measures of cannabis use (ever using during study period, any use, past week frequency) were associated with having detectable viral loads, though CD4 counts were comparable across cannabis use frequencies [6]. Participants in our study had HIV virologic control, were insured patients recruited from primary care clinics, and may have had better treatment adherence and management of their HIV symptoms compared with those in other settings. Nevertheless, future work should continue to examine the relationship of HIV control to higher risk cannabis use.

### Limitations

Data were collected from PWH enrolled through primary care sites at KPNC. Findings may not generalize to other populations, including those receiving treatment in non-primary care settings, are uninsured, or residing in states with restrictive cannabis use policies. More than half of participants were White and the majority were male. Nevertheless, the sample was recruited from a health system that is representative of the insured population of Northern California. Finally, the present analyses were limited to cross-sectional associations between HIV factors and cannabis use, and comments about causality cannot be made. Further research is needed to examine potential effects of HIV-related factors on changes in cannabis use over time.

## Conclusion

This study aimed to identify CUD risk factors among PWH identified through systematic digital screening in primary care. PWH who were Black, younger, and had elevated anxiety and tobacco use had increased odds of being at higher risk for CUD. Efforts should be made to continue monitoring mental health and substance use behaviors and address age-and race-based disparities in cannabis use impacts among PWH.

## Figures and Tables

**Table 1:** Characteristics of people with HIV reporting past 3-month cannabis use through screening in primary care

	*Complete sample*	*Sample by TAPS Cannabis Score*	
	*N* = 978	Higher Risk for CUD [Cannabis TAPS≥2] (*n* = 350)	*Any Use* [Cannabis TAPS =1] (*n* = 628)
Age in years, N (%)			
17–30	77 (7.9)	38 (10.9)	39 (6.2)
31–40	139 (14.2)	49 (14.0)	90 (14.3)
41–50	153 (15.6)	60 (17.1)	93 (14.8)
51–60	306 (31.3)	84 (24.0)	222 (35.4)
61–70	232 (23.7)	89 (25.4)	143 (22.8)
71+	71 (7.3)	30 (8.6)	41 (6.5)
Sex, N (%)			
Male	920 (94.1)	593 (94.4)	327 (93.4)
Female	58 (5.9)	35 (5.6)	23 (6.6)
Race/Ethnicity, N (%)			
Asian	49 (5.0)	12 (3.4)	37 (60.8)
Non-Hispanic Black	192 (19.6)	96 (27.4)	96 (15.3)
Hispanic	133 (13.6)	43 (12.3)	90 (14.3)
Non-Hispanic White	570 (58.3)	188 (53.7)	382 (60.8)
Other/UK	34 (3.5)	11 (3.1)	23 (3.7)
NDI quartile, N (%)			
1 (least deprived)	312 (31.9)	99 (28.3)	213 (33.9)
2	228 (23.3)	75 (21.4)	153 (24.4)
3	216 (22.1)	78 (22.3)	138 (22.0)
4 (most deprived)	222 (22.7)	98 (28.0)	124 (19.8)
Charlson Comorbidity Index, mean (SD)	0.82 (1.5)	0.97 (1.7)	0.74 (1.4)
PHQ-9 (≥10), N (%)	155 (15.9)	68 (19.4)	87 (13.9)
GAD-2 (≥3), N (%)	158 (16.2)	78 (22.3)	80 (12.7)
Higher Risk Tobacco Use, N (%)	101 (10.3)	56 (84.0)	45 (7.2)
Higher Risk Alcohol Use, N (%)	272 (27.8)	100 (28.6)	172 (27.4)
Prior Depression Diagnosis, N (%)	198 (20.3)	82 (22.4)	116 (18.5)
Prior Anxiety Diagnosis, N (%)	181 (18.5)	76 (21.7)	105 (16.7)
Prior SUD Diagnosis, N (%)	188 (19.2)	88 (25.1)	100 (15.9)
HIV RNA Log10, mean (SD)	1.8 (0.2)	1.8 (0.6)	1.8 (0.5)
CD4+ T cells/*u*L, mean (SD)	666.1 (290.2)	675.2 (326.0)	661.0 (268.1)

**Note:** Tobacco, Alcohol, Prescription medication, and other Substance use (TAPS) Tool administered through an electronic screen prior to or during clinic visits. Primary outcome was cannabis score (range 0–3), categorized as non- higher risk use (1) and higher risk for cannabis use disorder (Higher risk for CUD) (≥2). NDI = Neighborhood Deprivation Index; PHQ-9 = Patient Health Questionnaire, positive screen with a score of 10 or greater; GAD-2 = General Anxiety Disorder, positive screen with score of 3 or greater; HIV RNA log 10 transformed; SUD= Substance Use Disorder; Higher Risk Tobacco Use and Higher Risk Alcohol Use defined as Tobacco or Alcohol TAPS Score =≥2.

**Table 2:** Demographic and clinical predictors of scoring at higher risk for cannabis use disorder among people with HIV

	Unadjusted		Multivariable	
Predictors	OR	95% CI	OR	95% CI
Age				
17–30	**1.79**	**(1.02, 3.15)**	1.74	(0.96, 3.14)
31–40 (Reference)				
41–50	1.19	(0.74, 1.91)	1.25	(0.75, 2.06)
51–60	0.70	(0.45, 1.07)	0.76	(0.48, 1.21)
61–70	1.14	(0.74, 1.77)	1.33	(0.81, 2.19)
71+	1.34	(0.75, 2.41)	1.57	(0.82, 3.02)
Race/ethnicity				
White (Reference)				
Hispanic	0.97	(0.00, 0.65)	1.03	0.66, 1.61
Black	**2.03**	**(1.46, 2.83)**	**1.84**	**1.29, 2.63**
Asian	0.66	(1.37, 0.24)	0.64	0.32, 1.31
Other/Unknown	0.97	(0.14, 0.46)	0.84	0.38, 1.86
Sex (Male)	0.83	(0.49, 1.44)		
Charlson Comorbidity Index	**1.10**	**(1.01, 1.20)**	1.07	0.97, 1.18
GAD-2 (3+)	**1.96**	**(1.39, 2.77)**	**1.91**	**1.22, 2.98**
PHQ-9 (10+)	**1.50**	**(1.06, 2.12)**	0.93	0.59, 1.46
Higher Risk Tobacco Use	**2.47**	**(1.63, 3.74)**	**2.27**	**1.47, 3.51**
Higher Risk Alcohol Use	1.06	(0.79, 1.42)	-	-
Prior Depression Diagnosis	1.19	(0.70, 2.05)	-	**-**
Prior Anxiety Diagnosis	1.45	(0.84, 2.49)	-	**-**
Prior SUD Diagnosis	1.50	(0.80, 2.79)	-	**-**
CD4+ T cells/ *u*L (per 100 cells)	1.02	(0.97, 1.07)	-	**-**
HIV RNA (per 1 log)	1.20	(0.94, 1.53)	-	**-**

**Note:** Unadjusted regression models predicting higher risk cannabis use (Cannabis TAPS≥2); significant variables from significant unadjusted models entered in multivariable regression model (age, race, GAD, higher risk tobacco use, Charlson Comorbidity Index, PHQ-9)

Bolded values indicate significance at *p* <.05; OR = Odds ratio; CI = Confidence Interval; GAD = General Anxiety Disorder; PHQ-9 = Patient Health Questionnaire; SUD = Substance Use Disorder; HIV RNA, log 10 transformed; Higher Risk Tobacco Use and Higher Risk Alcohol Use defined as Tobacco and Alcohol TAPS Score≥2; PHQ-9 defined as ≥10; GAD-2 score defined as ≥3.

## Data Availability

The datasets generated and/or analysed during the current study are not publicly available.
